# Supplementary material for: DMRforPairs: identifying Differentially Methylated Regions between unique samples using array based methylation profiles
Source: BMC Bioinformatics. 2014 May 15;15:141. doi: 10.1186/1471-2105-15-141 (PMC4046028; doi:10.1186/1471-2105-15-141)
Supplement: Additional file 2 — DMRforPairs output for the comparison of A431-MCF7 and NA17018-NA17105. Please start from the HTML files in each folder. Available via the BMC Bioinformatics website. [file 1471-2105-15-141-S2.zip › 1394847754114233_MOESM2_ESM/A431_MCF7/figures/9814.pdf]

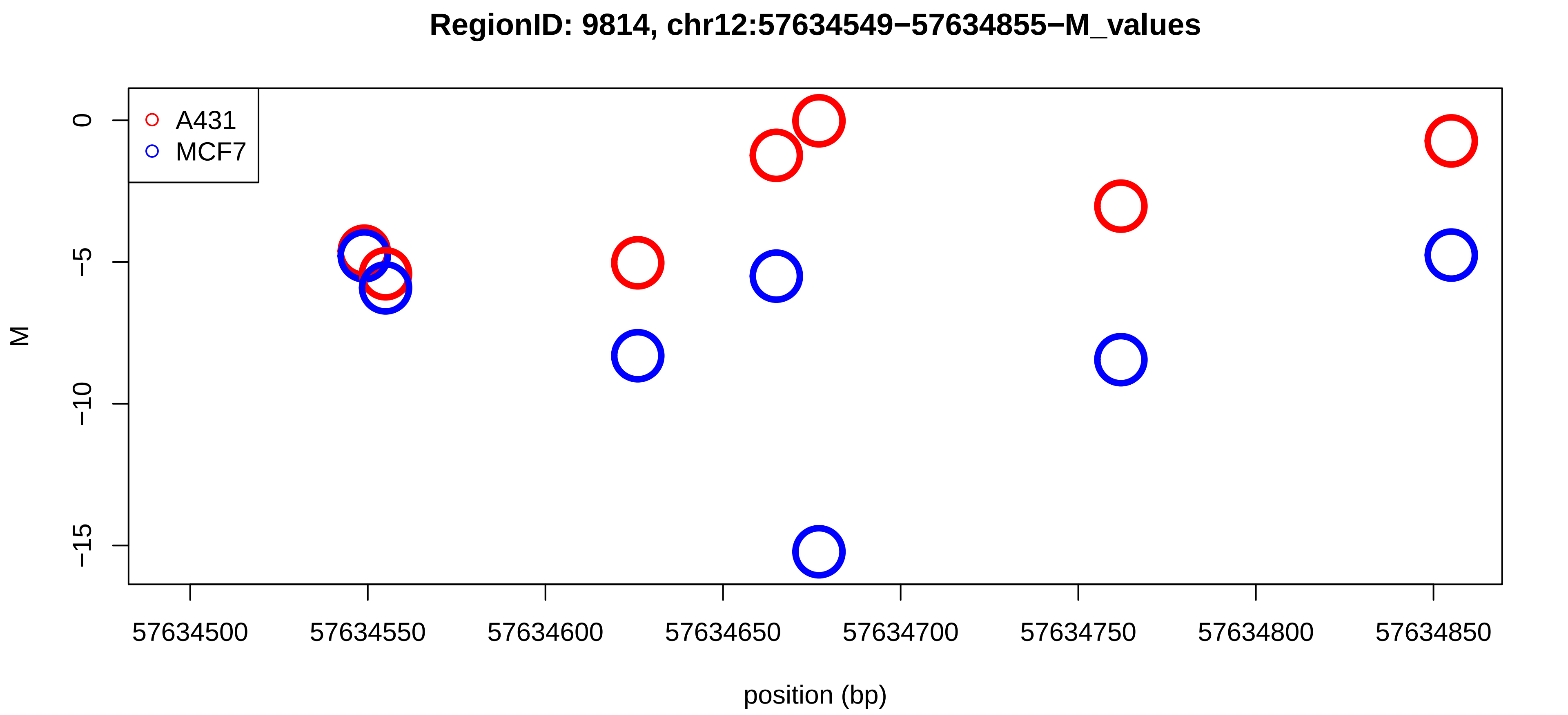

RegionID: 9814, chr12:57634549−57634855−M_values
A431
MCF7
M
0
−5
−10
−15
57634500
57634550
57634600
57634650
57634700
57634750
57634800
57634850
position (bp)

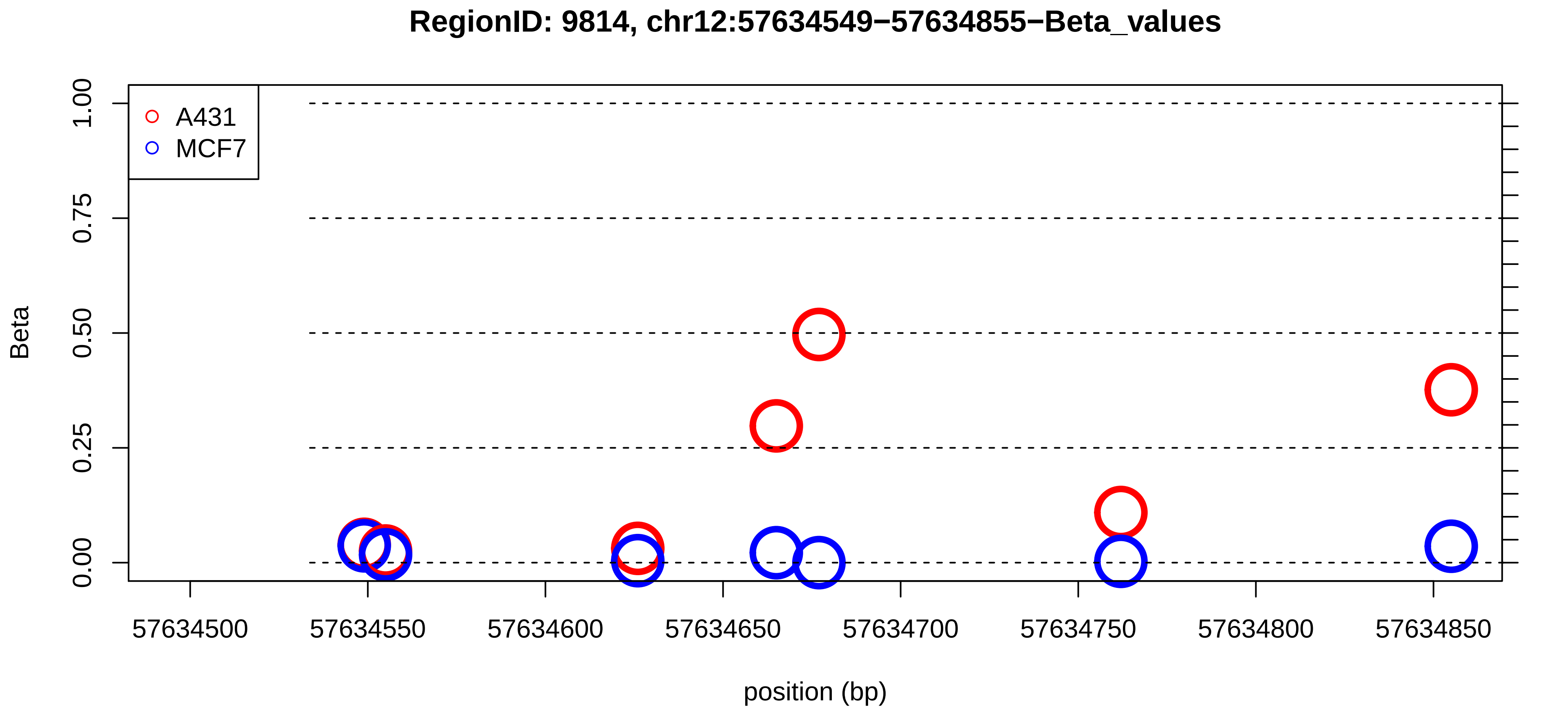

RegionID: 9814, chr12:57634549−57634855−Beta_values
A431
MCF7
Beta
1.00
0.75
0.50
0.25
0.00
57634500
57634550
57634600
57634650
57634700
57634750
57634800
57634850
position (bp)

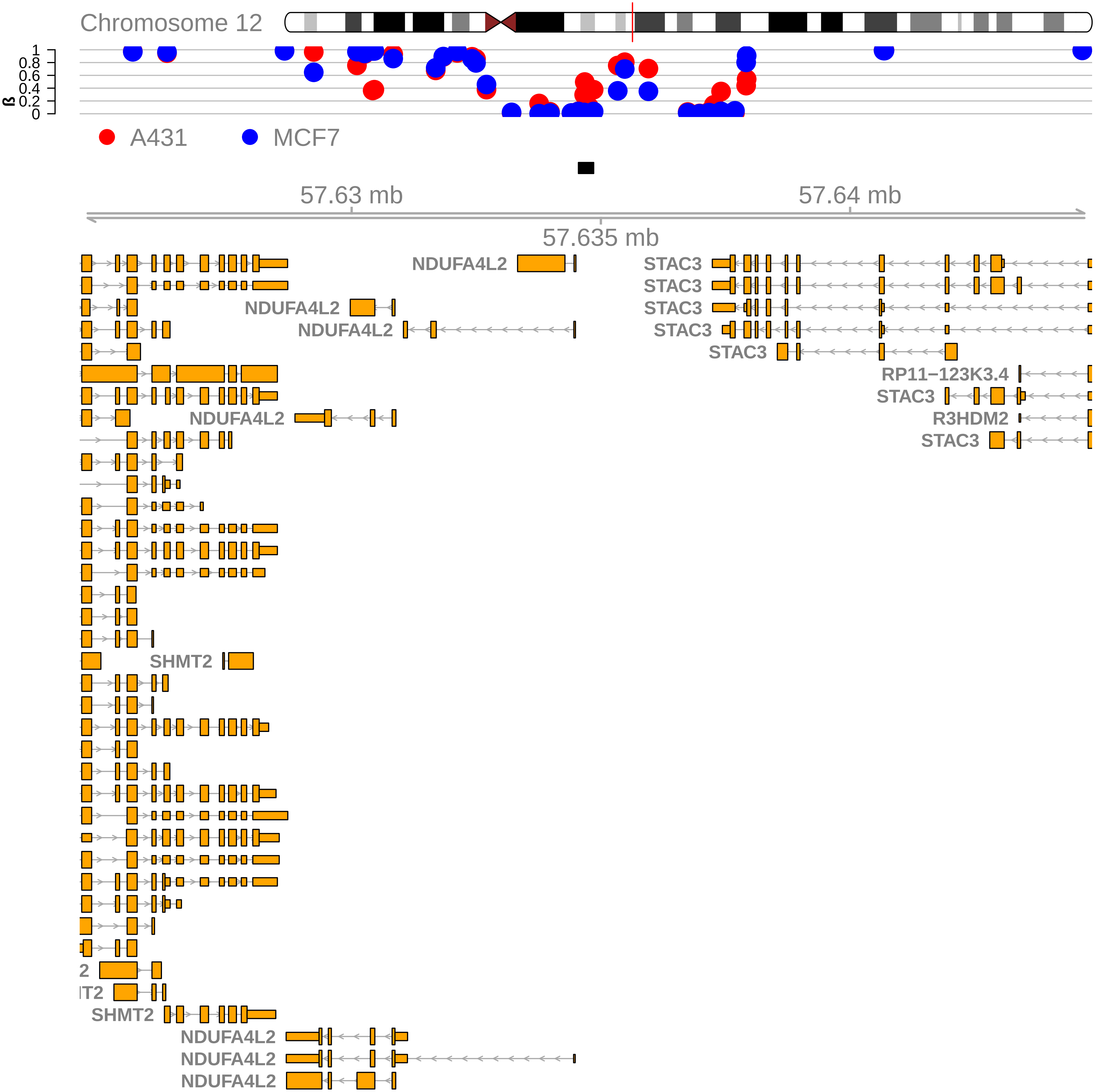

Chromosome 12
β
1
0.8
0.6
0.4
0.2
0
A431
MCF7
57.63 mb
57.635 mb
57.64 mb
NDUFA4L2
STAC3
STAC3
NDUFA4L2
STAC3
NDUFA4L2
STAC3
STAC3
RP11−123K3.4
STAC3
NDUFA4L2
R3HDM2
STAC3
SHMT2
SHMT2
NDUFA4L2
NDUFA4L2
NDUFA4L2
